# Supplementary material for: Learning curve and surgical outcome of robotic assisted colorectal surgery with ERAS program
Source: Sci Rep. 2022 Nov 29;12:20566. doi: 10.1038/s41598-022-24665-w (PMC9709162; doi:10.1038/s41598-022-24665-w)
Supplement: Supplementary file 1 — Supplementary Information 1. [file 41598_2022_24665_MOESM1_ESM.zip › LSC Raw data-Ñ[▒K/148/H_ñw╝╨░O▒Kñσ.pdf]

| 正式摘要(電子簽章：00000023408l444) | | |
|---|---|---|
| W52 / W52　☐英文標題(注意:無英文姓名-請補病人基本檔!) | ☐【讀取】憑證 | |

# 臺中榮民總醫院

## 出院病歷摘要

轉入號 :　　　　　　　　轉出號 :

[redacted]

| | |
|---|---|
| (8)流水編號 | |
| (9)入院日期 | 111/01/04 (2022) 直腸外科 W52 - 121 |
| (10)轉科日期 | |
| (11)出院日期 | 111/01/10 (2022)　住院天數計 6 日 |

### 診斷

- 入院 :malignant tumor of cecum
- 出院 :malignant tumor of cecum

### 主訴

- Tarry stool for 1 year

### 病史

- This is a 73 years old male patient denied underlying disease.

  According to the patient, he suffered from anemia and tarry stool since 1 year ago. He had visited other Hospital where anemia was noticed, and EGD was done without significant findings. Further survey was suggested but the patient refused. About 6 months later, DOE was noticed lab data showed anemia. Thus, he received colonoscopy at other Hospital, showed Cecum cancer. Then, he visited our CRS OPD for second opinion. As mentioned above, this time he was admitted to our ward for scheduled operaiton.

### 體檢發現

- 
  - General appearance :
    - easy looking , conscious clear , without cardiopulmonary distress
    - Vital signs自動帶入date 體溫(BT) 脈搏(P) 呼吸(R) 血壓(BP) 身高(BH) 體重(BW) BMI
      20220104 36.4℃ 91次/分 18次/分 139/88mmHg 166.3cm 58.4kg 21.1
    - Vital signs: B.P. 139/88 mmHg , P.R. 91 /min , R.R. 18 /min , B.T. 36.4 ℃
  - HEENT :
    - Eyes: no anemic conjunctiva, no icteric sclera, normal EOM
    - ENT: no otorrhea, no rhinorrhea, no erythematous mucosa, no injected tonsil, no deformity
  - Neck: supple, freely movable, trachea midline,no deformity, no engorged jugular vein , no palpable thyroid or lymph node enlargement
  - Chest:
    - Inspection: Grossly normal, symmetric and fully expansion, no accessory muscle used, no operation scar, no spider angiomata, no gynecomastia
    - Palpation: no palpable mass or nodule, no subcutaneous emphysema, no tenderness
    - Ausculation: clear breathing sound
  - Heart:
    - Palpation: no thrill, no heave
    - Percussion: normal heart size
    - Ausculation: regular heart beat, normal S1, S2, no gallop, no murmur
  - Extremities: No deformity, full ranged of motion without limitation, no clubbing finger or nail cyanosis, no pitting edema, no ulcer or pigmentation, no obvious skin lesion, no varicose vein, no bone, joint or muscle tenderness

**CRS CONDITION**

- 
  - Karnofsky performance status: (100) Normal, no complaints, no evidence of disease
  - PE of Abdomen:
  - CEA: 17.96 ng/ml
  - Imaging Studies:
  - Colonoscope report: .
  - Figures:
    1. colon & rectum

## 手術日期及方法

- 手術日期及方法20220106 1.Laparoscopy ureterolithotomy
  20220106 1. Laparoscopic right hemicolectomy with side to end anastmosis by EEA 29mm (small bowel end to T colon side) + GIA 75mm-blue *1
  2. Ileo-colic artery and middle colic right branch was ligated via Hemo-clips.
  3. Take down of hepatic flexure : (+)
  4. Cut margin: proximal (ileum) >10 cm, (colon) >10 cm
  5. Repair of mesentery defect : Nil.
  6. we consult GU doctor, left ureteral stone was remove under laparoscope and left D-J was inserted

## 住院治療經過

- This is a 73 years old male patient denied underlying disease.
  This time he was admitted to our ward for scheduled operation, and we arrange 1. ERAS pre-OP protocol 2.ICG liver function test 3. Blood transfusion: RBC 2U 4. PET for operation. The operation was done on 1/6. On 1/7 (POD1) patient's wound pain is tolerable and his vital sign stable lab data : WBC:8830 RBC:4.17 HGB:10.3 HCT:33.4 MCV:80.1 PLATE:187 NEUT:83.8 LYM:10.2 MONO:5.8 EOSINO:0.1 BASO:0.1 ALB:3.2 BILT:1.42 BILD:0.39 ALKP:88 GOT:21 GPT:9 NA:137 K:4.0 CL:105 CA:8.0 BUN:12 CREAT:1.28 LIPA:22 GFR:58.55 On 1/8 patient had no fever but he complained urinary retention , then urinary analysis was arranged, the outcome of U/A revealed bacteriuria r/o UTI DRE revealed mild enlarged prostate so the Tamsulosin and bethanechol was orderd. Patient's condition is stable and wound condition looked well so he was allowed to discharge on 1/10.

## 合併症

## 一般檢查

## 特殊檢查

## 病理報告

## 其它

## 出院時情況

- 3.改門診治療

## 出院指示

## 出院計劃

主治醫師_2062F 蔣鋒帆

正式摘要(電子簽章：**000000234081444)**

W52 / W52

doctorRank:B
